# Correlating biological activity to thermo-structural analysis of the interaction of CTX with synthetic models of macrophage membranes

**DOI:** 10.1038/s41598-021-02552-0

**Published:** 2021-12-09

**Authors:** Luciana de Araújo Pimenta, Evandro L. Duarte, Gabriel S. Vignoli Muniz, Kerly Fernanda Mesquita Pasqualoto, Marcos Roberto de Mattos Fontes, M. Teresa Lamy, Sandra Coccuzzo Sampaio

**Affiliations:** 1 Pathophysiology Laboratory, Instituto Butantan, Av. Vital Brazil, 1500, Butantã, SP 05503-900 Brazil; 2 Department of Pharmacology, Institute of Biomedical Sciences, University of São Paulo, Av. Lineu Prestes, 1524, Butantã, SP 05508-900 Brazil; 3 Institute of Physics, University of São Paulo, R. Do Matão, 1371, Butantã, SP 05508-090 Brazil; 4 Alchemy Inovação, Pesquisa e Desenvolvimento Ltda., Technology-Based Business Incubator of São Paulo IPEN/USP – Cietec, Av. Lineu Prestes, 2242 – Building D, Butantã, SP 05508-000 Brazil; 5 Department of Biophysics and Pharmacology, Institute of Biosciences, Paulista State University, Prof. Dr Antonio Celso Wagner Zanin, 250, Botucatu, SP 18618-689 Brazil

**Keywords:** Membrane structure and assembly, Phagocytes

## Abstract

The important pharmacological actions of Crotoxin (CTX) on macrophages, the main toxin in the venom of *Crotalus durissus terrificus*, and its important participation in the control of different pathophysiological processes, have been demonstrated. The biological activities performed by macrophages are related to signaling mediated by receptors expressed on the membrane surface of these cells or opening and closing of ion channels, generation of membrane curvature and pore formation. In the present work, the interaction of the CTX complex with the cell membrane of macrophages is studied, both using biological cells and synthetic lipid membranes to monitor structural alterations induced by the protein. Here we show that CTX can penetrate THP-1 cells and induce pores only in anionic lipid model membranes, suggesting that a possible access pathway for CTX to the cell is via lipids with anionic polar heads. Considering that the selectivity of the lipid composition varies in different tissues and organs of the human body, the thermostructural studies presented here are extremely important to open new investigations on the biological activities of CTX in different biological systems.

## Introduction

Crotoxin (CTX) was the first protein purified from the rattlesnake, *Crotalus durissus terrificus* venom, which was isolated by Slotta and Fraenkel-Conrat^1^. Subsequently, Fraenkel-Conrat and Singer^2^ described its composition, being a heterodimeric β-neurotoxin, formed by a non-covalent association of a non-toxic acid subunit, Crotoxin A (CA or Crotapotin) and a basic subunit called Crotoxin B (CB) with phospholipase A_2_ (PLA_2_) activity. The subunit CB^3^ and CTX^4^ were solved, revealing the importance of its individual isoforms and, more recently, further details of in solution CTX characteristics were revealed^5^. The CTX is the main toxin in the rattlesnake venom, corresponding to around 60% of the total proteome of the venom^6,7^. Its molecular mass is 24 to 26 kDa and its isoelectric point is approximately 4.7^5,8^.

Several studies have shown that CTX has several biological activities, such as neurotoxicity, myotoxicity, nephrotoxicity and cardiotoxicity. In addition, this toxin has antitumor, anti-inflammatory, antiviral and immunomodulatory actions^9–12^. Regarding the effects on the immune system, it was evidenced that crotalic venom or CTX have a suppressive role on the humoral and cellular immune response^11,13–17^.

Regarding the inflammatory response, Nunes and collaborators^9^ showed that the long-term inhibitory action of CTX on the vascular and cellular components of the inflammatory response, induced by carrageenan, was more effective when compared to classic anti-inflammatories, thus characterizing the anti-inflammatory effect suggested for the total venom^18^. The anti-inflammatory action of CTX is associated with a decrease in the expression of adhesion molecules in cytokine secretion neutrophils and an increase in the secretion of resolving lipid mediators, such as lipoxin A_4_ and 15-Epi-LXA_4_, with important involvement of receptors for formyl peptide on leukocyte migration^19^. In addition, this toxin inhibits the phagocytosis capacity of both neutrophils^10^ and macrophages^15^. In this sense, in macrophages, dualism in the action of this toxin was evidenced, since it was observed both inhibition of some functional parameters, such as spreading and phagocytosis^15^, and stimulation of the respiratory burst (generation of oxygen peroxide), the generation of nitric oxide and the glucose and glutamine metabolism of these cells.

Still regarding macrophages, the biological activities performed by these cells, such as growth, differentiation, activation, recognition, endocytosis, migration and secretion, are processes related to signaling mediated by receptors expressed on the surface of the membrane of these cells. In addition to receptors, these biological activities also involve processes such as opening and closing ion channels, generating membrane curvature and the formation of pores^20–26^.

It is noteworthy that in a cell membrane, phospholipids and sphingolipids are not uniformly distributed and not only impart unique dielectric and permeability properties to the bilayer, but also determine the partition and folding of intrinsic proteins^27^. Glycerophospholipids represent approximately 70% of the total lipid content of mammalian cells and, among phospholipids, phosphatidylcholine (PC) is the most prevalent and accounts for 40% to 50% of the total. Grando and collaborators^28^ demonstrated that the incorporation of different fatty acids into PC by peritoneal macrophages can be an important pathway for the alteration of macrophage activity. Still on macrophages and PC, Petkevicius and collaborators^29^ demonstrated an important relationship between regulation and synthesis of PC-lipids, macrophage turnover and membrane fluidity in an inflammation model in cases of obesity. Although the phospholipid headgroup phosphatidylserine (PS) is more commonly found in the internal leaflet of the plasma membrane, under physiological conditions, externalized PS functions as a dominant immunosuppressive signal that promotes tolerance and prevents local and systemic immune activation. Pathologically, externalized PS is used by countless viruses, microorganisms and parasites to facilitate infection and, in many cases, establish the latency of the infection. And even under pathological conditions, PS signaling is highly deregulated in the tumor microenvironment and in autoimmune diseases^30^. In macrophages, PS is functionally significant for phagocytosis of target cells that express PS^31^. Regarding the hydrocarbon chains, on the peritoneal macrophage cell membrane Felippe and collaborators^32^ demonstrated that the main total neutral lipids of these cells that are part of its composition are palmitic acid (34%), oleic acid (26%), and stearic acid (19%). The distinct lipid composition gives characteristic properties of thickness, permeability and fluidity to the organelle membranes.

Taken together, data from the literature have demonstrated the important pharmacological actions of CTX on the cells of the immune system, in particular macrophages, and their important participation in the control of different pathophysiological processes. Therefore, elucidating how this toxin interacts with different molecules in the membrane, such as receptors, channels and lipids of these cells, will favor, in an important way, the understanding of the mechanisms involved with their biological activities.

**Figure 1:**
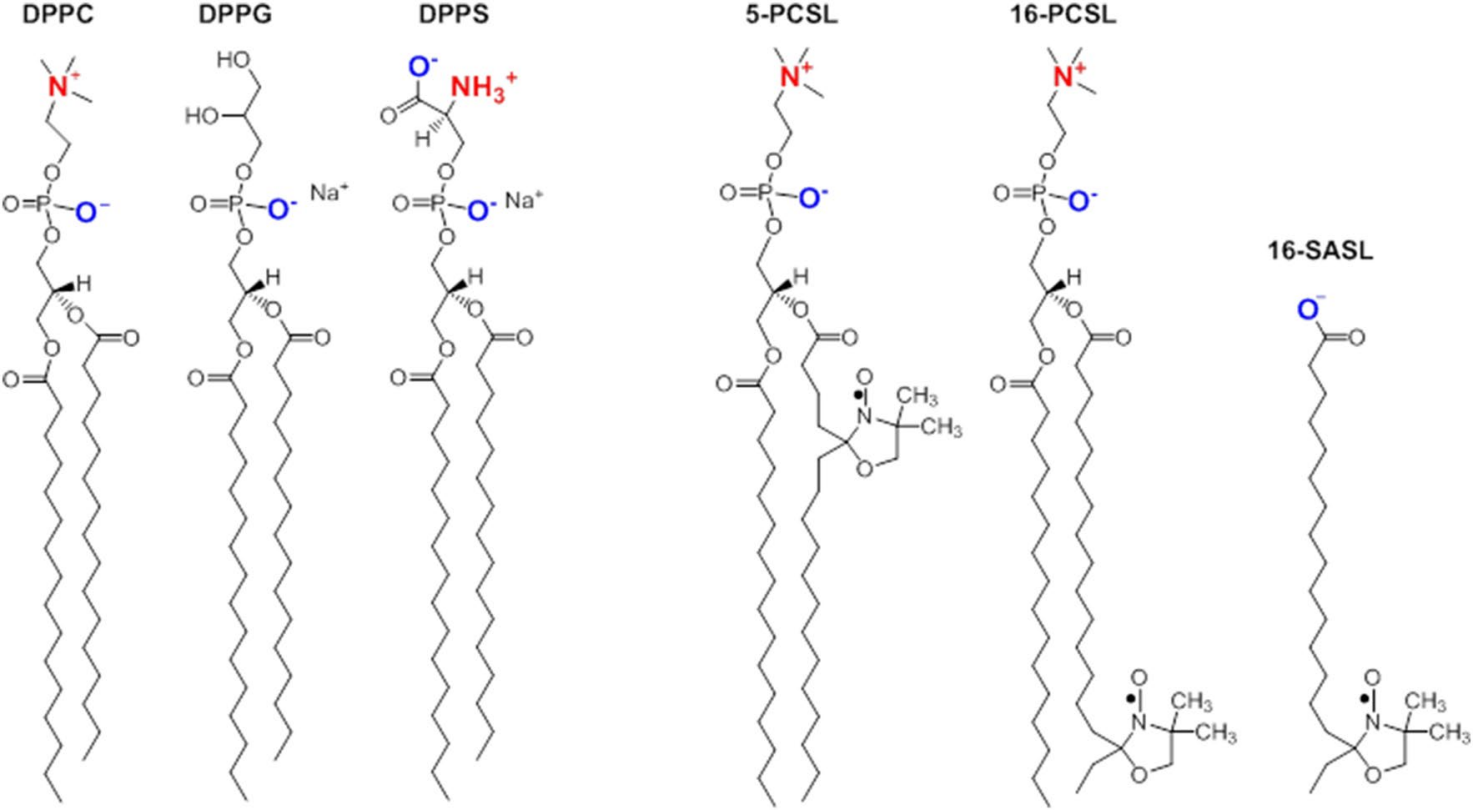
Chemical structures of the lipids and spin labels used. In red and blue, positive and negative groups, respectively.

In this context, the aim of this work was to study the effect of CTX on the structure of model membranes composed of synthetic phospholipids with zwitterionic and anionic head groups. Therefore, we report here, our investigations on the thermo-structural behavior of lipid membranes (Fig. 1) of 1,2-dipalmitoyl-*sn*-glycero-3-phosphocholine (DPPC), 1,2-dipalmitoyl-*sn*-glycero-3-phospho-(*1’*-*rac*-glycerol) (sodium salt) (DPPG), and 1,2-dipalmitoyl-*sn*-glycero-3-phospho-L-serine (sodium salt) (DPPS), in the absence and presence of the CTX. Varying the temperature, the synthetic membranes were monitored at their gel and fluid phases, which mimic more and less packed regions in a natural membrane, respectively. The liposomes were characterized by differential scanning calorimetry (DSC), electron spin resonance (ESR) of spin labels incorporated into the bilayer, and the assessment of membrane pore formation, through the measurements of the leakage of entrapped carboxyfluorescein (CF), a fluorescent dye. Moreover, we demonstrated that, in vitro, CTX can penetrate THP-1 cells.

## Results and discussions

### CTX penetrates THP-1 cells

**Figure 2:**
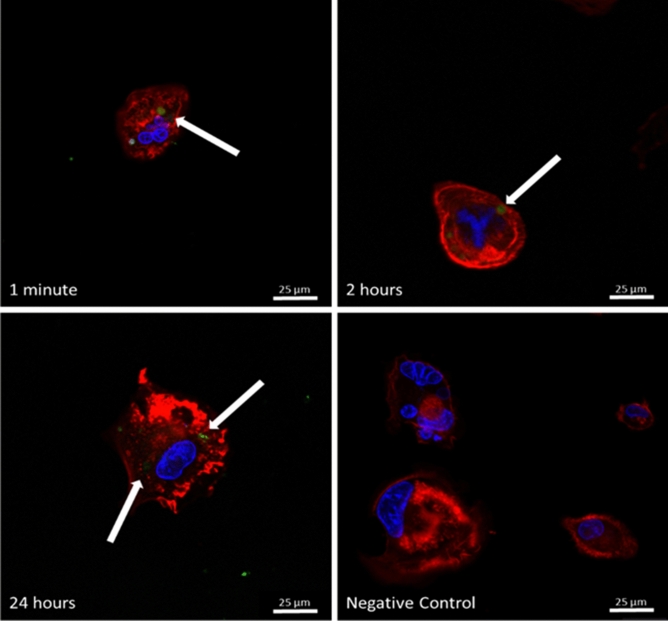
Confocal microscope images of fluorescein isothiocyanate (FITC)-labeled CTX (green, indicated by arrows) in THP-1 cells with 1 min, 2 h and 24 h of incubation. In red represents the actin filaments (stained with rhodamine phalloidin) and in blue the cell nucleus (stained with DAPI—4′,6-diamidino-2-phenylindole). The scale bar corresponds to 25 µm. The images were analyzed in confocal microscopy under the Leica TCS SP8-Confocal Microscope at 40 × magnification.

Figure 2 shows the fluorescence microscopy images obtained from CTX labeled with fluorescein isothiocyanate (FITC) at different incubation times in THP-1 cells. It is possible to observe in red the actin filaments (stained with rhodamine phalloidin) and in blue the cell nucleus (stained with DAPI—4′,6-diamidino-2-phenylindole).

In order to confirm the interaction of CTX with the membrane and its penetration into the cell, the fluorescence microscopy image assay with CTX FITC-labeled in THP-1 cells was performed. As shown in Fig. 2, the toxin can be found inside the cell after 1 min, 2 h and 24 h incubation and after washing with culture medium. It is important to mention that a negative control was performed to verify the absence of toxin and autofluorescence in all evaluated periods.

The rapid entry of toxin into the cell, evidenced by CTX FITC-labeled, corroborates data from Sampaio and collaborators^15^, who showed that the inhibitory action of toxin on the zymozan particle phagocytosis process was observed after 5 min of incubation. In this sense, previous studies have shown that internalization of peptides obtained from snake venoms occurs within minutes^33,34^. This behavior was also observed for crotamine, one of the toxins also present in *C. d. terrificus* venom^33,35^. Interestingly, these authors observed the entry of this peptide into different cells, including human primary fibroblasts, lymphoblastic cells, murine embryonic cells, and endothelial cells.

### CTX induces pores in anionic membranes

Carboxyfluorescein leakage assay is a powerful method to study pore formation on lipid vesicles caused by exogenous molecules^36,37^. Briefly, the fluorophore carboxyfluorescein (CF) at high concentration, encapsulated in liposomes, displays no fluorescence due to self-quenching. After the addition of CTX in the liposome dispersion, if the protein causes membrane modification such that the liposome becomes permeable to CF, the fluorophore would get diluted into the bulk, hence its fluorescence would increase, due to the decrease of the self-quenching effect.

**Figure 3:**
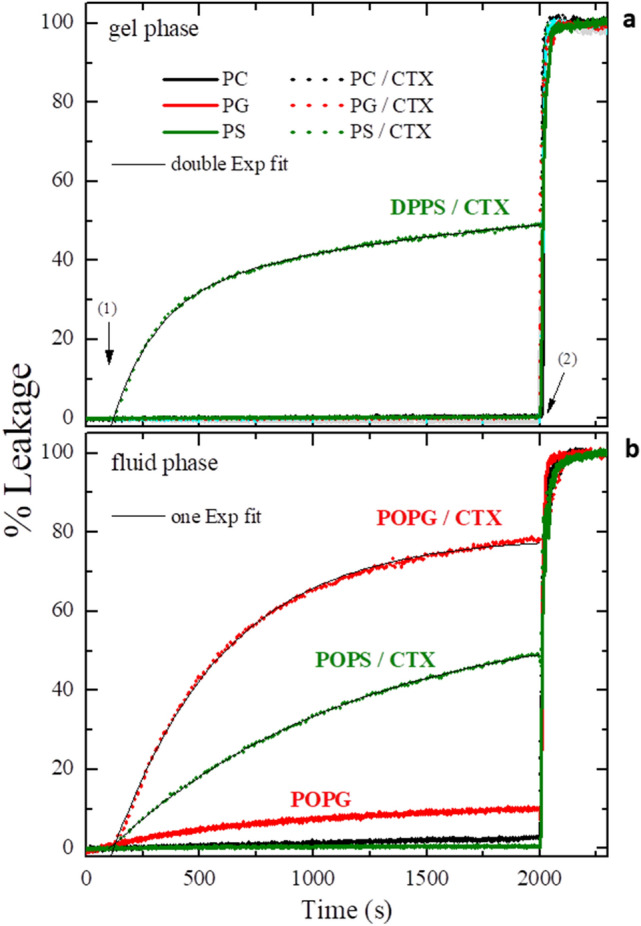
Kinetics of carboxyfluorescein (CF) leakage through LUVs composed of 100 µM of DPPC, DPPG and DPPS, in the gel phase, and POPC, POPG and POPS in the fluid phase, in absence and presence of 0.1 mol% of CTX relative to the lipid concentration (see Eq. (2) in MM section). All experiments were performed at 25 °C. Arrow (1) indicates the moment the protein was injected, and arrow (2) the moment of surfactant was added. Black thin solid lines represent the best fittings using Eq. (1).

Figure 3 displays the leakage kinetics performed with different lipid vesicles, DPPC, DPPG and DPPS, in the gel phase, and 1-palmitoyl-2-oleoyl-*sn*-glycero-3-phosphocholine (POPC), 1-palmitoyl-2-oleoyl-*sn*-glycero-3-phospho-(1'-rac-glycerol—sodium salt) (POPG) and 1-palmitoyl-2-oleoyl-*sn*-glycero-3-phospho-L-serine (sodium salt) (POPS), in the fluid phase, with and without the addition of 0.1 mol% CTX, in relation to the used lipid concentration, 100 µM. The experiments were carried out at 25 °C. The lipids, POPC, POPG and POPS were used to mimic DPPC, DPPG and DPPS in the fluid phase, respectively (see, Materials and methods section, for details).

As expected, all lipids without the addition of protein, in the gel phase, presented very low CF fluorescent emission along the acquisition window of 30 min, meaning a CF leakage of less than 1% for DPPC (black solid line), DPPG (red solid line) and DPPS (olive solid line), as shown in Fig. 3a. After 2000s, the detergent Triton X-100 was added and the total % of leakage was induced (indicated in Fig. 3a by arrow 2). With the addition of 0.1 mol% of CTX at the 100^th^ second of measurement (indicated in Fig. 3 by arrow 1), the permeability of the DPPC / CTX and DPPG / CTX was not altered, compared with pure lipid vesicles. However, the DPPS / CTX mixture (Fig. 3a—olive dotted line) evinced an increase of CF leakage, reaching around 47% leakage along the acquisition window.

In the membrane fluid phase shown in the Fig. 3b (at 25 °C), the lipids POPC and POPS, without addition of CTX presented negligible spontaneous leakage (less than 1%), and the lipid POPG presented an acceptable spontaneous leakage around 10% in 30 min of measurement, as previously observed^36^. Under the conditions studied here, with the addition of 0.1 mol% CTX, no change was observed with the zwitterionic lipid POPC. On the other hand, a strong leakage was detected with POPG / CTX and POPS / CTX systems, around 80% and 50% of leakage, respectively, at the end of the period of measurement (Fig. 3b).

Therefore, CTX does not cause CF leakage through neither packed (gel) or loose (fluid) PC lipid bilayers, but can damage PS (gel and fluid) and PG (fluid) bilayers, as CF can leek out of the vesicles. Considering that CF is a relatively big charged molecule, the opened bilayer pores, either permanent or temporary, should be quite large. It is important to note that CTX, at the concentration used here, does not destroy the bilayers, as vesicles of similar size are detected before and after CTX incubation (Table SM1).

For a clearer understanding of the leakage processes, the kinetics profiles were analyzed considering exponential processes Hence, the decay curves (Fig. 3) were fit with the minimum number of terms displayed in Eq. (1):

1 $$ \% {\text{ Lekeage}} \left( t \right) = A_{1} \left( {1 - e^{{{\raise0.7ex\hbox{${ - t}$} \!\mathord{\left/ {\vphantom {{ - t} {\tau_{1} }}}\right.\kern-\nulldelimiterspace} \!\lower0.7ex\hbox{${\tau_{1} }$}}}} } \right) + A_{2} \left( {1 - e^{{{\raise0.7ex\hbox{${ - t}$} \!\mathord{\left/ {\vphantom {{ - t} {\tau_{2} }}}\right.\kern-\nulldelimiterspace} \!\lower0.7ex\hbox{${\tau_{2} }$}}}} } \right) + A_{3} \left( {1 - e^{{{\raise0.7ex\hbox{${ - t}$} \!\mathord{\left/ {\vphantom {{ - t} {\tau_{3} }}}\right.\kern-\nulldelimiterspace} \!\lower0.7ex\hbox{${\tau_{3} }$}}}} } \right) + \ldots $$

Accordingly, the CF leakage through gel vesicles of DPPS (Fig. 3a) could not be fit by just one exponential, but could be well fit by two exponentials (see black thin solid line in Fig. 3a). Hence the leakage of CF through gel DPPS vesicles seems to be related to two processes (1 and 2), characterized by two different time constants, τ_1_ = 190 s and τ_2_ = 1700s, where A_1_ and A_2_ are the percentages of CF leakage at the end of the processes 1 and 2. (see Table 1).

**Table 1:** CF kinetic parameters from the best fittings of the kinetics in Fig. 3 with Eq. 2.

Sample	A_1_ (%)	τ_1_ (s)	A_2_ (%)	τ_2_ (s)	_□_^2^
DPPS (gel)	31.0	19 ✕ 10	27.2	172 ✕ 10	0.998
DPPS (gel)	47.0	37 ✕ 10	–	–	0.987
POPS (fluid)	59.7	109 ✕ 10	–	–	0.999
POPG (fluid)	80.1	53 ✕ 10	–	–	0.997

Total leakage percentage of CF and time constant from process 1, A_1_ and τ_1_, respectively, and process 2, A_2_ and τ_2_, respectively, and reduced chi-square, □^2^.

The leakage through the fluid phases of POPG and POPS could be well fit with Eq. (1) using just one exponential (see black thin solid lines in Fig. 3b), with quite large time constants, around 1000 s and 500 s, for PS and PG respectively, showing that the action of the protein on lipid membranes is relatively slow.

It is interesting to note that, none of the leakage assays achieve 100% leakage (see values of A_T_ = A_1_ + A_2_ in Table 1), which would be expected if the CTX molecules would be able to visit all the vesicles, that is, if they would be in thermodynamic equilibrium between the aqueous medium and the membranes. Moreover, also remarkable, is that though through different mechanisms, the total leakage through gel DPPS membranes (A_1_ + A_2_ ~ 58%) is similar to that through fluid POPS (A_1_ + A_2_ ~ 60%) (Table 1). That is something to be noted, but will not be discussed here, as it is out of the scope of the present work, being a controversial issue in the literature (see for instance, references 35 and 37, and discussions therein).

### CTX changes the thermal behavior of anionic membranes

The thermal phase transition of a lipid bilayer depends on lipid-lipid interaction, and can be very sensitive to the presence of an exogenous molecule that interacts with the bilayer, informing about structural characteristics of molecule-lipid interaction.

**Figure 4:**
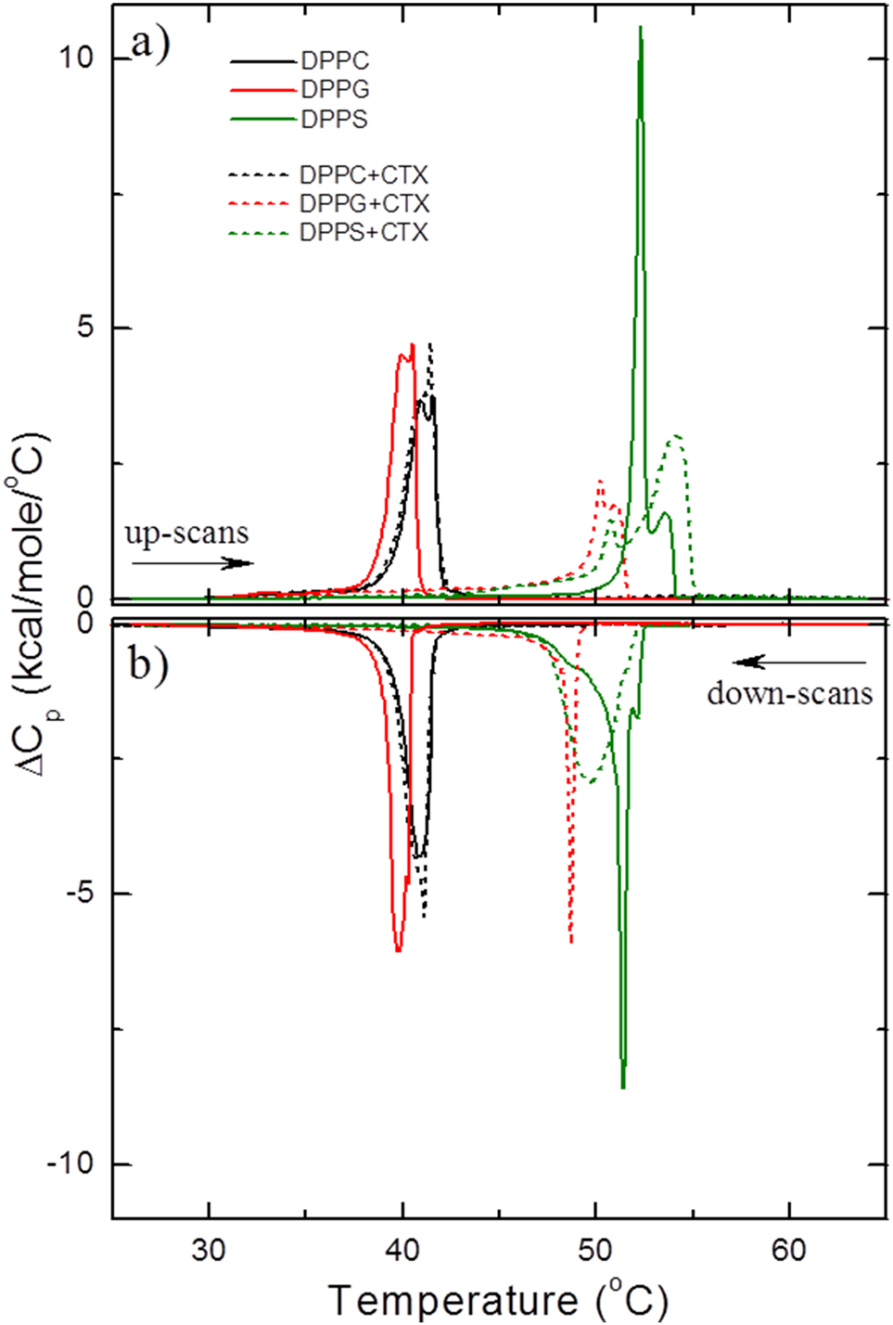
Typical excess heat capacity (ΔC_p_) profiles of 5 mM DPPC (black), DPPG (red) and DPPS (olive), in the absence (solid line) and presence (dotted line) of 0.1 mol% CTX relative to the lipid concentration: (**a**) heating and (**b**) cooling cycles.

DSC thermograms of extruded LUVs of 5 mM DPPC, DPPG and DPPS, in PBS buffer, in the absence and presence of 0.1 mol% of CTX, relative to the lipid concentration, are shown in Fig. 4. Measurements were carried out in both up- (endothermal cycle—Fig. 4a) and down (exothermal cycle—Fig. 4b) scans, in order to study the effect caused by the protein interaction on the reversibility of the lipid bilayer phase transition.

Up-scans for the pure extruded lipid vesicles used here display a thermal behavior characterized by a very smooth pre-transition followed by a relatively thin peak typical of the main gel-fluid transition (at *T*_*m*_) of LUVs (Fig. 4)^38^. For pure DPPC, DPPG and DPPS bilayers, the gel-fluid transition occurs at the temperatures of 41.5 ± 0.1 °C, 40.5 ± 0.1 °C, and 52.2 ± 0.1 °C, respectively, as expected^39^. The enthalpy variations, ΔH, and the width at half maximum, ΔT_½_, the latter associated with the transition cooperativity, for the three lipid membranes, are shown in Table 2. The values are in good agreement with the literature^40–43^. Moreover, DPPC and DPPG LUVs display a quite reversible main transition, whereas DPPS shows a small hysteresis (Fig. 4, and see *T*_*m*_ values for up and down scans in Table 2).

**Table 2:** Thermal parameters calculated form the calorimetric data in Fig. 4.

Samples	Up scan			Down scan		
	*T*_m_ (°C)	ΔH (kcal/mol)	ΔT_1/2_ (°C)	*T*_m_ (°C)	ΔH (kcal/mol)	ΔT_1/2_ (°C)
DPPC	41.5 (1)	8.5 (5)	1.6 (2)	41.1 (2)	8.3 (6)	1.4 (2)
DPPC + CTX	41.4 (1)	9.0 (3)	1.7 (1)	41.1 (2)	9.0 (2)	1.4 (1)
DPPG	40.5 (1)	8 (1)	1.3 (1)	39.7 (2)	8.6 (6)	1.1 (1)
DPPG + CTX	49.8 (3)	7 (1)	1.1 (5)	48.5 (2)	5 (1)	0.29 (2)
DPPS	52.2 (1)	8.8 (2)	0.42 (3)	51.3 (1)	9.0 (5)	0.4 (1)
DPPS + CTX	54.1 (1)	10 (1)	3 (1)	49.6 (1)	8.7 (3)	2.7 (1)

Mail transition temperature (Tm). Enthalpy variation (H), and main transition width at half maximum (T1/2). For the endothermic (up scan) and exothermic (down scan) cycles. The estimated standard deviations refer to the analysis of two different samples of the same system.

In the presence of CTX, no significant variation of the DPPC thermal behavior was observed (see Fig. 4 and Table 2). Together with the results discussed above, considering CF leakage assays, this is a strong indication that the protein does not cause any effect on the structure of DPPC vesicles, possibly remaining in buffer solution without partitioning into the zwitterionic membrane.

Very different thermal behaviors were observed for lipids with anionic head groups in the presence of the protein. DPPG main transition in the presence of 0.1 mol% CTX (Fig. 4a) occurs at T_m_ = 49.8 ± 0.3 °C, a huge shift of about + 9.3 °C as compared with pure DPPG membranes. Other thermodynamic parameters, ΔH and ΔT_½_ , do not change much, apart from a considerable increase in the cooperativity of the down-scan process for DPPG + CTX membranes (Table 2).

Hence, it seems that, somehow, the interaction of CTX with the DPPG membrane stabilizes the lipid gel phase, causing a shift of the main transition to higher temperatures. As it was shown earlier (Fig. 3), the protein, under the experimental conditions used here, does not cause any leakage in DPPG vesicles at 25 °C (gel phase). Those two results together suggest that CTX, in gel DPPG membranes, is electrostatically interacting at the surface of the vesicles, decreasing the PG¯–PG¯ repulsive interaction, hence increasing lipid–lipid packing.

Different from DPPG, for DPPS, the addition of CTX does not shift T_m_ much, but causes a remarkable loss of lipid–lipid cooperativity on the main thermal transition of the bilayer (Fig. 4a), with ΔT_½_ increasing from around 0.4 °C to 3 °C, for both scans, up and down (Table 2). Interestingly, DPPS is the only lipid studied here in which CTX induced leakage in its gel phase membrane (Fig. 3a). Hence, CTX seems to strongly interact with PS, including packed PS bilayers, disrupting them.

### Spin labels detect structural alterations caused on gel membranes by the presence of CTX in anionic dispersions

**Figure 5:**
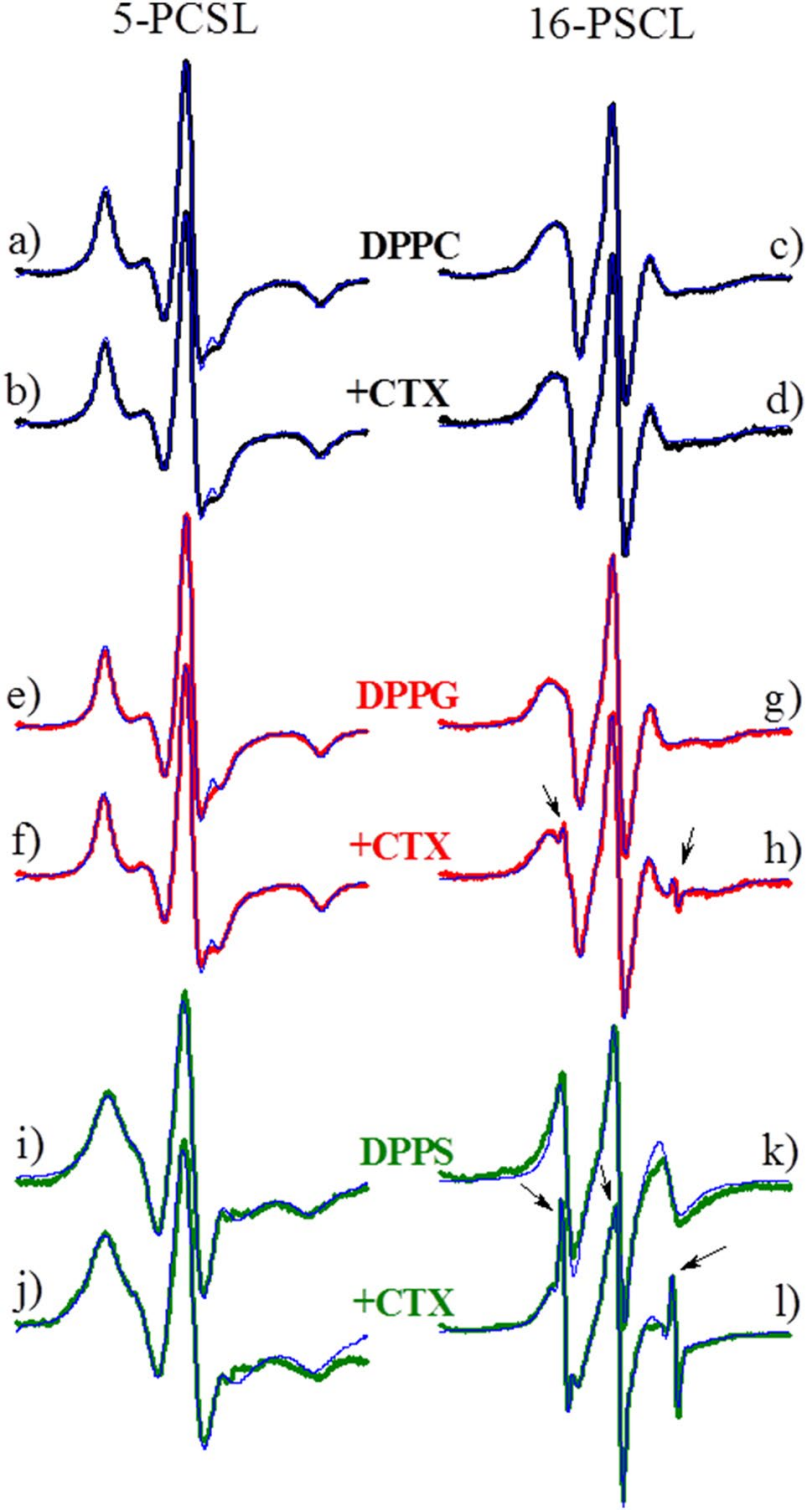
Typical ESR spectra of 5-PCSL and 16-PCSL spin labels incorporated into the gel phase of pure lipid bilayers of DPPC (**a**, **c**, black lines), DPPG (**e**, **g**, red lines) and DPPS (**i**, **k**, olive lines), and the mixtures of 0.1 mol% CTX relative to the lipid concentration of DPPC (**b**, **d**, black lines), DPPG (**f**, **h**, red lines) and DPPS (**j**, **l**, olive lines). The spectra were acquired at 20 °C, and its total width is 100 G. Blue lines correspond to the best fittings of the experimental spectra by the theoretical simulations (see MM).

ESR spectra, and the best theoretical simulations, of 5-PCSL and 16-PCSL embedded into gel membranes (20 °C, see DSC scans in Fig. 4) of pure DPPC, DPPG and DPPS vesicles are compared with those acquired in the presence of the protein (Fig. 5).

The paramagnetic nitroxide at the probe 5-PCSL is sensitive to the fluidity/order of the bilayer close to the membrane surface, whereas 16-PCSL monitors the bilayer core (Fig. 1). Accordingly, the ESR spectra of 5-PCSL incorporated into the membranes studied here are typical of a spin label in a more anisotropic environment than that of 16-PCSL incorporated in the same membranes, as the latter probes the more fluid environment at the bilayer core.

**Figure 6:**
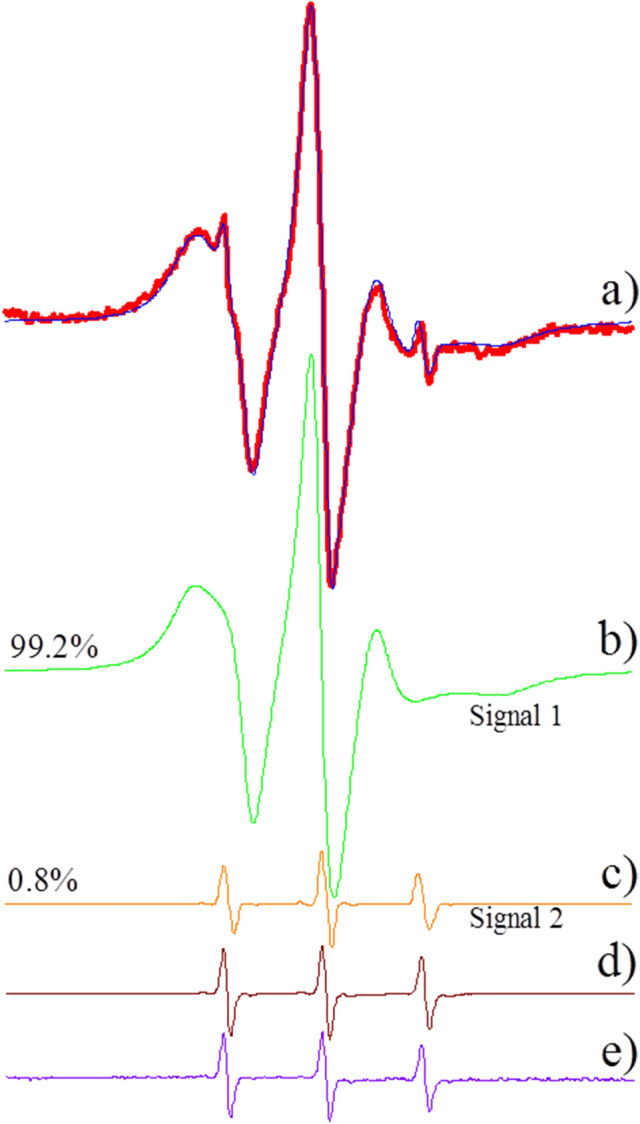
ESR experimental spectrum of the 16-PCSL incorporated in gel membrane of DPPG (5 mM) in the presence of 0.1 mol% CTX (**a**, red line), the same spectrum shown in Fig. 5 h. The blue line corresponds to the best fitting of the theoretical simulation on the experimental data considering two components: the anisotropic site (**b**, Signal 1, green line) and a highly isotropic site (**c**, Signal 2, orange line). The proportion of the two components are indicated. The theoretical mobile component is very similar to the experimental spectrum obtained for 16-PCSL in solution in presence of the Crotoxin (**d**, wine line) and that of the pure probe 16-SASL in buffer PBS (**e**, violet line). The total spectra width is 100G.

The possible structural changes caused by CTX on the membranes of DPPC, DPPG and DPPS were carefully analyzed through spectral simulations (see Material and Methods, for details). However, before discussing those changes, we would like to point out a very unusual result found with dispersions containing CTX and DPPG or DPPS in the gel phase: the clear presence of two coexisting ESR signals. Namely, different from the other ESR spectra shown in Fig. 5, the ESR signals of 16-PCSL in DPPG + CTX (Fig. 5h) and DPPS + CTX (Fig. 5l) could not be theoretically simulated as just one ESR signal: two ESR signals were necessary for the fitting, one more anisotropic, typical of spin labels in gel membranes (Fig. 6b, named Signal 1 here) and another one quite isotropic, usually found with spin labels tumbling nearly freely in solution (Fig. 6c, named Signal 2 here).

To try to rationalize the presence of those two different ESR signals, the following experiments were performed. A dried film of 16-PCSL was hydrated with buffer, following the same experimental procedure described for the preparation of lipid vesicles (see MM for details). As expected, no ESR signal was observed, as 16-PCSL is not soluble in water medium. However, we found that an ESR signal very similar to that highly isotropic Signal 2 (see Fig. 6c and d) appeared when the 16-PCSL film was hydrated with a buffer solution containing CTX, at the same concentrations used for the experiments with lipid membranes. So, CTX alone, in solution, incubated with a dried film of 16-PCSL, hence in contact with 16-PCSL in the film, gives rise to this mobile signal (Signal 2), similar to what happens in DPPG + CTX and DPPS + CTX dispersions, where 16-PCSL is incorporated in the lipid membranes. Interestingly, that does not happen with CTX in DPPC dispersions, where the spin label is also inside the lipid membrane. (Fig. 6 analyzes the ESR spectrum obtained with DPPG + CTX. See Fig. SM1 for a similar analysis with DPPS + CTX). Therefore, comparing with the results obtained with DSC (Fig. 4), where no effect of CTX on DPPC membranes was observed, we can conclude that CTX needs to be in contact with the lipid membrane, where 16-PCSL is incorporated, to give rise to this highly isotropic ESR signal (Signal 2, Fig. 6c).

Different from the phospholipid label 16-PCSL, the stearic acid spin label 16-SASL (Fig. 1) is aqueous soluble, and gives an ESR signal quite similar to Signal 2, the highly isotropic ESR signal (see comparisons in Fig. 6c, d and e). Hence, the results described above could be rationalized considering that CTX, when in contact with 16-PCSL, can lyse the phospholipid spin label yielding an aqueous soluble paramagnetic probe, similar to 16-SASL. Important to note that the percentage of spin labels that undergo lysis is very low, being 0.8% for DPPG + CTX (Fig. 6c) and 5.5% for DPPS + CTX (Fig. SM1c). Also, important to note that CTX lyses 16-PCSL but does not lyse 5-PCSL. Considering that both probes, 5- and 16-PCSL, are phospholipids, the possible lysis by CTX deserves further investigation, out of the scope of the present work.

As previously mentioned, we used ESR spectral simulations to obtain parameters that provide us with information about the dynamics, order and polarity of the different lipid membranes studied, in the absence and presence of Crotoxin, at the regions probed by 5- and 16-PCSL. Important to mention that the spectra shown in Fig. 5, for 5-PCSL in DPPS (i) and DPPS + CTX (j) membranes, were obtained after the subtraction of a very broad band attributed to spin–spin interaction, as shown in Fig. SM2. That was necessary, and somehow expected, due to the known low solubility of phospholipid spin labels in the gel phase of DPPS membranes^44^.

**Figure 7:**
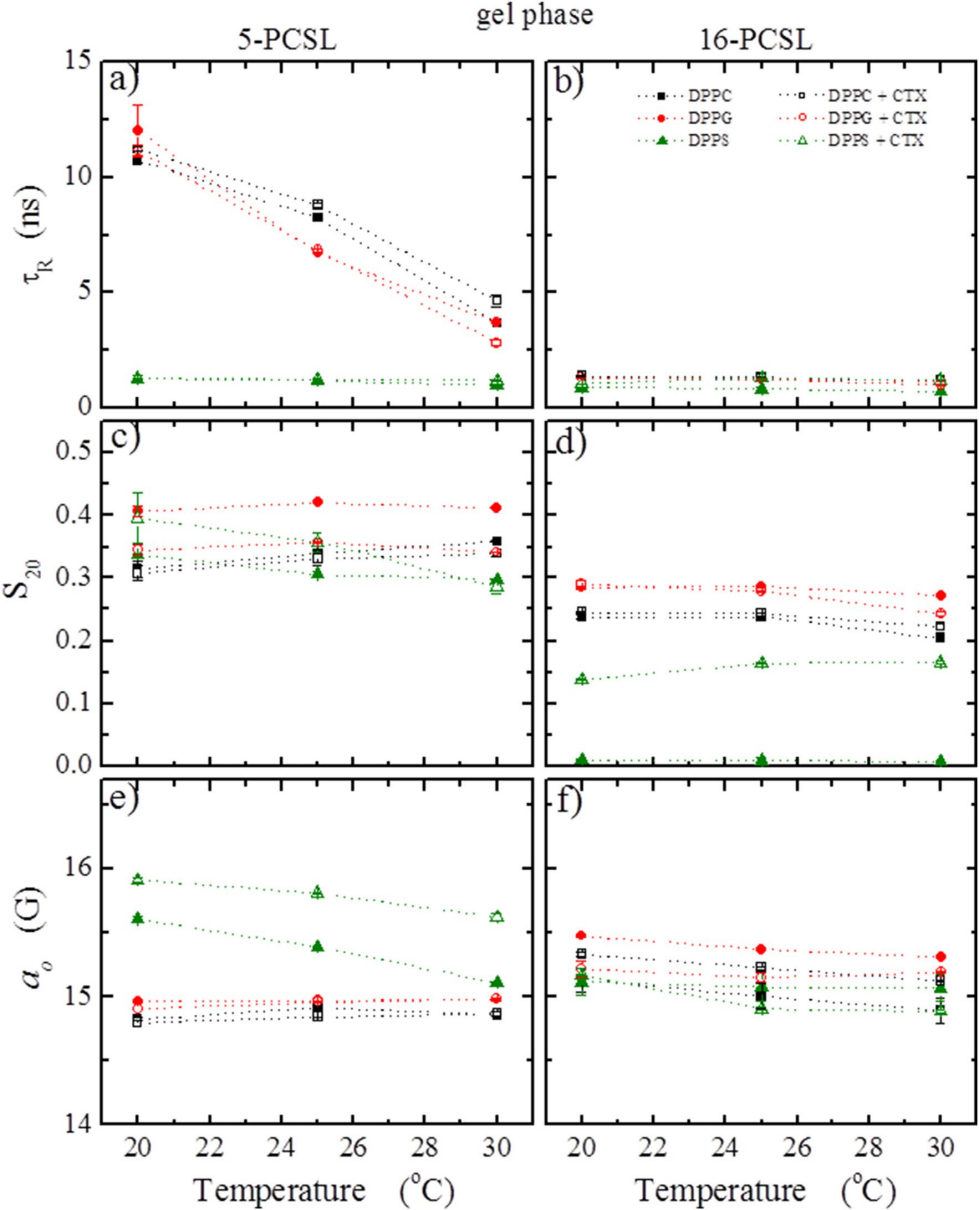
Temperature dependence of the parameters obtained from the best theoretical simulations of the spectra in Fig. 5: (**a** and **b**) average rotational correlation time *τ*_*R*_, (**c** and **d**) order parameter *S*_*20*_, and (**e** and **f**) isotropic hyperfine splitting *a*_*o*_. The left and right columns correspond to the parameters obtained from spectra of the spin labels 5- and 16-PCSL, respectively, inserted in pure lipids (solid symbols) and in the mixtures of lipid/protein (open symbols), at the lipids gel phase.

Figure 7 compares the values of the main parameters yielded by the theoretical simulations of the ESR signals of 5- and 16-PCSL incorporated into gel lipid vesicles, in the absence (open symbols) and in the presence (closed symbols) of CTX. The average rotational correlation time, *τ*_*R*_ (a and b), is related to the mobility of the spin labels in the membranes, the order parameter, *S*_*20*_ (c and d), depends on the local microscopic order of the probe into the bilayer, and the isotropic hyperfine splitting, *a*_*o*_ (e and f) reflects the micro-polarity felt by the nitroxide in the probes (see MM for additional explanation). For 16-PCSL in DPPG and DPPS vesicles, the simulated ESR signals where those that correspond to the label incorporated in the membranes, Signal 1, hence after the subtraction of the highly mobile Signal 2 from the experimental spectra (Fig. 6 and Fig. SM1). Theoretical simulations are represented in blue lines over the experimental spectra in Fig. 5.

In accord with DSC data (Fig. 4), no change was observed with CTX in DPPC dispersions (black symbols in Fig. 7), confirming that the protein does not interact with DPPC gel membranes.

For gel membranes of DPPG, CTX does not alter the mobility of the spin labels (*τ*_*R*_, Fig. 7a, b), but causes a slight decrease of the order of the bilayer (*S*_*20*_) around the 5th carbon atom (Fig. 7c), and a decrease of the bilayer polarity (*a*_*o*_) at its core (Fig. 7f).

The gel lipid membrane found to be most affected by CTX was that of DPPS. The protein considerably changes the structure of the membrane, causing a significant increase on its order at the membrane core (*S*_*20*_ in Fig. 7d), and also increasing the DPPS bilayer polarity around the 5^th^ carbon atom position.

It is interesting to compare these results with those obtained with the kinetics of CF leakage through the gel lipid vesicles (Fig. 3a): DPPS was the only vesicle studied here to become permeable to CF in the presence of CTX. Hence this observed increase in the bilayer polarity caused by CTX in gel DPPS membranes (Fig. 7c) are possibly related to the opening of aqueous pores in the membrane.

### Spin labels detect strong structural alterations on fluid anionic membranes caused by CTX

**Figure 8:**
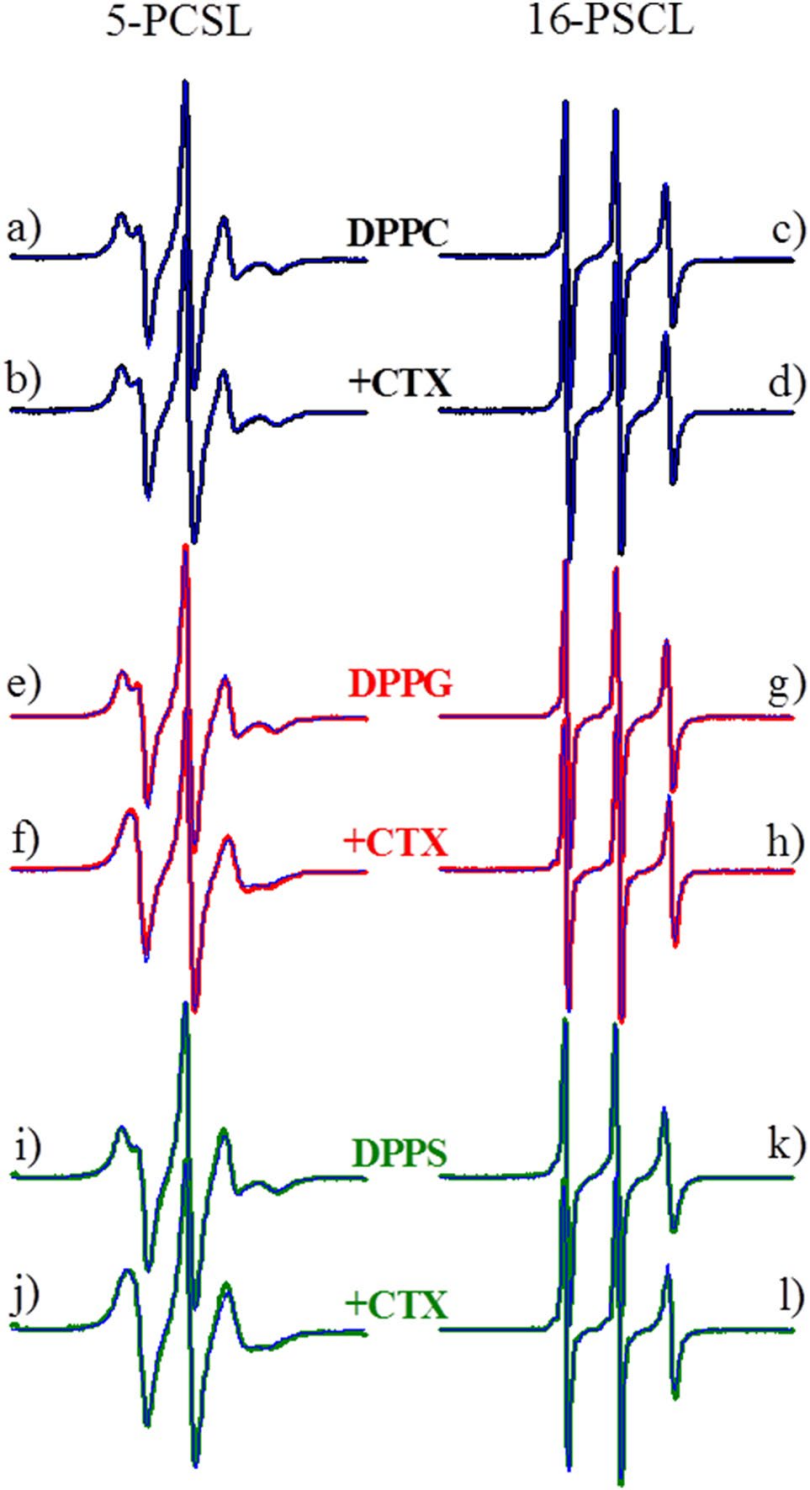
Typical ESR spectra of 5-PCSL and 16-PCSL spin labels incorporated into the fluid phase of pure lipid bilayers of DPPC (**a**, **c**, black lines), DPPG (**e**, **g**, red lines) and DPPS (**i**, **k**, olive lines), and the mixtures of 0.1 mol% CTX relative to the lipid concentration of DPPC (**b**, **d**, black lines), DPPG (**f**, **h**, red lines) and DPPS (**j**, **l**, olive lines). The spectra were acquired at 60 °C, and its total width is 100 G. Blue lines correspond to the best fittings of the experimental spectra by the theoretical simulations (see MM).

**Figure 9:**
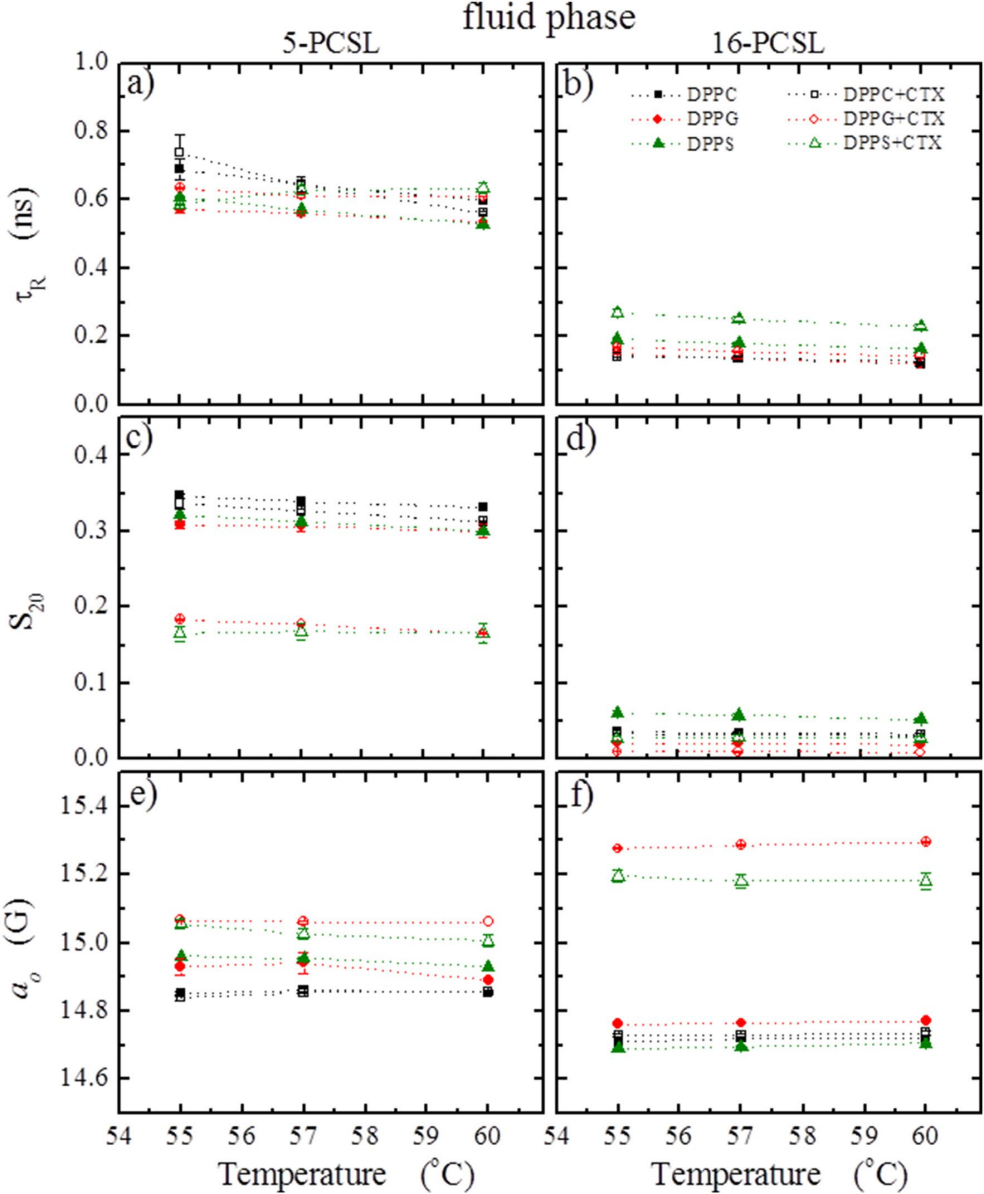
Temperature dependence of the parameters obtained from the best theoretical simulations of the spectra in Fig. 8 (**a** and **b**) average rotational correlation time *τ*_*R*_, (**c** and **d**) order parameter *S*_*20*_, and (**e** and **f**) isotropic hyperfine splitting *a*_*o*_. The left and right columns correspond to the parameters obtained from spectra of the spin labels 5- and 16-PCSL, respectively, inserted in pure lipids (solid symbols) and in the mixtures of lipid / protein (open symbols), at the lipid fluid phase.

The ESR spectra in Fig. 8 is a typical ESR spectra of spin labels in fluid membranes, and they all could be well simulated as only one ESR signal, and the parameters of the best fittings are shown in Fig. 9.

Similar to that found for the DPPC gel phase, the ESR parameters show no structural alterations caused by CTX on fluid DPPC membranes (black symbols in Fig. 9), again, strongly supporting that the protein does not interact with DPPC vesicles.

CTX was found to significantly affect the structure of fluid membranes of both DPPG and DPPS. The most striking effects were the significant decrease in the organization of the membranes close to their surface, around the 5^th^ carbon atom (decrease on *S*_*20*_ , Fig. 9c), and the huge increase of the polarity of the bilayers at their core (increase of *a*_*o*_, Fig. 9f). Apart from those changes, CTX also caused a small increase on the membrane polarity at the 5^th^ carbon atom in both fluid DPPG and DPPS membranes (Fig. 9e), and a small decrease of the membrane order at the core of the bilayers (Fig. 9d).

Again, the above ESR results are in line with those obtained with the kinetics of CF leakage through the fluid lipid vesicles (Fig. 3b), and the formation of permanent or transient pores: both POPG and POPS vesicles become permeable to CF in the presence of CTX.

## Conclusions

With the cell culture assay, using confocal microscope images, we observed that the Crotoxin (CTX) isolated from South American rattlesnake venom interacts with the membrane and penetrates macrophage cells.

Thermal and structural studies with model membranes, composed of lipids only, showed that 0.1 mol% CTX, relative to the lipid concentration, does not cause any effect on zwitterionic membranes of PC, and causes important changes on negatively charged lipid bilayers of PG and PS. Therefore, it is possible to conclude that CTX does not interact with zwitterionic PC membranes, under the conditions studied here.

CTX strongly disrupts gel and fluid DPPS membranes, causing the formation of pores on the membrane at both phases, as attested by carboxyfluorescein leakage assays (Fig. 3). The disruptions CTX causes on DPPS membranes are also attested by the broadening of the gel-fluid bilayer thermal transition (Fig. 4), the significant decrease on the bilayer order (Fig. 9) and the huge increase in the bilayer polarity (Fig. 9). The latter two conclusions drawn from spin labels incorporated into the membrane.

Interestingly, DSC shows that CTX interacts with DPPG membranes, but it stabilizes the gel phase, shifting the gel-fluid temperature to much higher values (Fig. 4). Accordingly, CTX does not induce pores on DPPG gel membranes (Fig. 3), and causes only small effects on DPPG gel bilayers, as monitored by spin labels (Fig. 7). However, when the DPPG lipids are more fluid, less packed, above DPPG Tm, CTX penetrates DPPG membrane, inducing the formation of bilayer pores, resulting in leakage of CF even faster than that observed in DPPS membranes (Fig. 3). In addition, spin labels show that CTX causes significant decrease on the bilayer order, and increase on the bilayer polarity of fluid DPPG membranes.

With regard to human cell charges, studies have shown that tumor cells display negative charged lipids on their external membrane. Hence it is interesting to correlate the data presented here with the biological activity of the CTX molecule, as several studies demonstrate its antitumor capacity, suggesting the interaction of CTX with negative lipid vesicles only^45^. Therefore, the results presented here suggest that one possible route of access of CTX to the cell (as shown in Fig. 2) is through lipids with anionic polar head groups.

Furthermore, considering that the selectivity of the lipid composition varies in different tissues and organs of the human body, thermo-structural studies are extremely important to understand the different activities described for CTX in real biological systems.

## Materials and methods

### Chemicals and reagents

The lipids and its chemical structures demonstrated in the scheme below, 1,2-dipalmitoyl-*sn*-glycero-3-phosphocholine (DPPC), 1,2-dipalmitoyl-*sn*-glycero-3-phospho-(*1’*-*rac*-glycerol) (sodium salt) (DPPG) and 1,2-dipalmitoyl-*sn*-glycero-3-phospho-L-serine (sodium salt) (DPPS), and spin labels (Fig. 1) 1-palmitoyl-2-stearoyl-(***n***-doxyl)-*sn*-glycero-3-phosphocholine (***n***-PCSL, ***n*** = 5 or 16) were supplied by Avanti Polar Lipids (Birmingham, AL, USA). Phosphate buffered saline, PBS, reagents (10 mM phosphate at physiological ionic strength with the addition of 137 mM of NaCl and 3 mM of KCl, pH 7.4), HEPES buffer (4-(2-hydroxyethyl)-1-piperazineethanesulfonic acid), EDTA (ethylenediaminetetraaceticacid disodium salt), CF (5(6)-carboxyfluorescein), glucose and Triton-X100 were purchased from Sigma Chemical Co. (St. Louis, MO, USA). Ultrapure water was used throughout.

### Crotoxin (CTX)

Lyophilised venom of *C. d. terrificus* was obtained from the Laboratory of Herpetology, Butantan Institute, São Paulo, Brazil, and stored at − 20 °C. Crude venom solution was subjected to anion-exchange chromatography as previously described by Rangel-Santos and collaborators^46^, using a Mono-Q HR 5/5 column in an FPLC system (Pharmacia, Uppsala, Sweden). The fractions (1 mL/min) were eluted using a linear gradient of NaCl (0–1 M in 50 mM Tris–HCl, pH 7.0). Three peaks (p1, p2 and p3) were obtained: p2 corresponded to the pure CTX fraction (about 60% of the crude venom); peaks 1 and 3 included the other CdtV toxins. Before pooling, the fractions containing CTX were tested for homogeneity by non-reducing sodium dodecyl sulphate–polyacrylamide gel electrophoresis (12.5%) and the phospholipase A_2_ activity was assessed by a colorimetric assay using a synthetic chromogenic substrate^8,46–48^.

### Liposome preparation

Lipid films were formed solubilizing powder lipids in chloroform solution, the sample was dried under a stream of N_2_ and left under reduced pressure for a minimum of 2 h to remove all traces of organic solvent. The liposome dispersion was prepared by the addition of buffer solution (either PBS or HEPES). The lipid dispersion was heated above the lipid phase transition, at 65 °C for 5 min, and stirred vigorously. This process was repeated 4 times, until a homogeneous and turbid solution was obtained. The dispersion was extruded 31 times through a polycarbonate membrane with 100 nm pores and above the lipid gel-fluid phase transition temperature, using a mini-extruder by Avanti Polar Lipids. At the end of the extrusion protocol, a translucent solution of large unilamellar vesicles (LUV) with approximately 100 nm diameter was obtained. When required, 0.1 mol% of CTX, relative to the total lipid concentration, was added directly to the extruded lipid dispersion, from a stock solution of CTX in PBS. Dynamic light scattering, DLS, was used in order to confirm the average diameter of the vesicles in the absence and presence of CTX (Table SM1).

For ESR experiments, 0.8 mol% 5-PCSL or 0.3 mol% 16-PCSL, relative to the total lipid concentration, were added to the lipid chloroform solution when preparing the lipid film as described before. All samples were used right after preparation.

### Differential scanning calorimetry (DSC)

DSC thermograms were obtained in a Microcal VP-DSC Microcalorimeter (Microcal Inc., Northampton, MA, USA) equipped with 500 µL twin total-fill cells. Lipid dispersions at a final lipid concentration of 5 mM in buffer PBS were heated from 20 up to 70 °C, at a scan rate of 20 °C/h. Scans were performed at least in duplicate. Baseline corrections and peak analysis were done using the Microcal Origin software with additional pack for calorimetry data analysis provided by Microcal. The thermodynamic parameters, enthalpy of the transition, ΔH, main gel-fluid phase transition temperature, *T*_*m*_, and width at half maximum, ΔT_1/2_, were obtained.

### Electron spin resonance (ESR) spectroscopy

ESR measurements at X-band (9.44 GHz) were performed with a Bruker EMX spectrometer, with a high sensitivity ER4119HS cavity. Lipid dispersions at a final lipid concentration of 5 mM in buffer PBS were placed in flame sealed capillary tubes. Field-modulation amplitude in the range of 1.0—2.0 G (depending on the line widths), sweep width of 100 G and microwave power of 13 mW were used. The temperature was controlled to about 0.1 °C with a Bruker BVT-2000 variable temperature device from 20 °C up to 70 °C, and monitored with a Fluke 51 K/J thermometer with a probe placed just above the cavity.

### ESR spectral simulations

ESR spectra display clear differences evidenced by their broadening, which are directly associated to the dynamics, order and polarity of the environment probed by the spin label^49,50^. For instance, spectra of spin labels incorporated at the gel and fluid phases of a bilayer are well characterized by an anisotropic or a more isotropic ESR signal, respectively. Beside this, the spectra are highly dependent on how deep in the bilayer the probes are found. Two spin labels were used in this work, the 5-PCSL and 16-PCSL (Fig. 1), where the paramagnetic nitroxide radical is located at the 5^th^ carbon of the hydrocarbon chain, close to the polar head, and at the 16^th^ carbon, close to the bilayer core, respectively. At the same experimental condition, the spectrum of 5-PCSL is found to be more anisotropic than that of 16-PCSL, indicating that the membrane is less packed/ordered at the core than close to the surface of the bilayer^51^. Theoretical simulations of the ESR spectra were performed using the computer program “MultComponent EPR fitting”, version 826, LabVIEW (National Instruments), developed by Christian Altenbach, and freely available for download at https://sites.google.com/site/altenbach/downloads. The polarity, ordering and rotational dynamic parameters were obtained through a nonlinear least-squares (NLLS) minimization algorithm, based on the stochastic Liouville equation developed by Freed and coworkers^52,53^. The MOMD model (which stands for microscopic order and macroscopic disorder)^54^ was considered in order to simulate the ESR spectra of spin labels incorporated into unilamellar vesicles. Since the parameters used have been extensively discussed in earlier publications^53,54^, only a few of the most relevant points for performing the simulations will be mentioned here.

Considering that the variations on the magnetic tensor, g-tensor, with the label local polarity is rather small, we kept constant the values of the g-tensor components g_xx_ = 2.0086 ± 5, g_yy_ = 2.0063 ± 3 and g_zz_ = 2.0023 ± 2, which are values usually applied for both spin labels 5- and 16-PCSL embedded in different lipids vesicles^53,55–57^.

The hyperfine splitting tensor was considered to be axial, *A*_*xx*_ = *A*_*yy*_ , and the *A*_*zz*_ values were allowed to vary within a reasonable interval^55,56^. The amount of water inside the bilayer may be dependent on the lipid phase and temperature. From the hyperfine splitting tensor components, *A*_*xx*_, *A*_*yy*_ and *A*_*zz*_, an isotropic hyperfine splitting can be calculated, *a*_*o*_ = (*A*_*xx*_ + *A*_*yy*_ + *A*_*zz*_)/3, which indicates the degree of polarity sensed by the probes in the bilayers.

The local microscopic order of the spin label in the lipid bilayer is characterized by the order parameter, *S*_*20*_, calculated from the best parameters obtained from the first term of the expansion of the ordering potential in generalized spherical harmonics (Schneider and Freed 1989). Thus, when all probe molecules are completely oriented parallel to the bilayer normal, *S*_*20*_ is equal to unity. On the other hand, *S*_*20*_ tends to zero when the spin labels rotate in a rapid isotropic motion^58^.

The dynamics of the spin label is characterized by the average rotational correlation time, *τ*_*R*_, calculated from the main components of the axially symmetric rotational diffusion tensor, *R*_//_ and *R*_⊥_. For the probes used here, 5- and 16-PCSL for example, they represent the rotational rates of the nitroxide moiety around axes parallel and perpendicular to the hydrocarbon chain, respectively. For the simulations, we assumed a Brownian rotational diffusion with a constant anisotropy N = *R*_***//***_ / *R*_⊥_ = 10 usually applied^54^. The average rotational diffusion rate is defined as $\overline{R}=\sqrt[3]{{R}_{\perp }^{2}\cdot {R}_{//}}$, and the average rotational correlation time, ${\tau }_{R}={\left(6\overline{R}\right)}^{-1}$.

All ESR data shown are means of at least two experiments, and the uncertainties are the standard deviations. When not shown, the uncertainty was found to be smaller than the symbol in the graph.

### Leakage assay

Following the similar LUV preparation protocol described above, the lipid film was hydrated to a final lipid concentration of 6 mM, in buffer (10 mM HEPES with 1 mM EDTA and 100 mM NaCl at pH 7.4) containing 50 mM carboxyfluorescein (CF). CF stock solution was prepared in HEPES buffer at pH 8.5, and after solubilization the sample pH was readjusted to 7.4 with HCl_._ The lipid dispersion was extruded 31 times, through a polycarbonate membrane with 100 nm pores and above the lipid gel-fluid phase transition temperature. To remove non entrapped CF, the suspension of LUVs was eluted through a Sephadex-G25 medium column with the gradual addition of the buffer, 10 mM HEPES pH 7.4 with 1 mM EDTA, 100 mM NaCl and 150 mM glucose, the latter added to the buffer to adjust the osmolarity inside and outside of the liposomes. Vesicles with entrapped CF (CF-LUVs) were collected in the void volume of the column. The precise final lipid concentration was determined by inorganic phosphate assay^59^. A final dilution was made using buffer 10 mM HEPES with 1 mM EDTA and 100 mM NaCl at pH 7.4, to obtain a lipid concentration of 100 µM. It is worth to note that as the inorganic phosphate assay was necessary for determining the lipid concentration precisely, the PBS buffer had to be replaced by HEPES buffer in this experiment.

Samples were placed in quartz cuvettes (10 × 10 mm, 2 ml) and the fluorescence emission measured with a fluorimeter (Varian Cary Eclipse, Santa Clara, CA), with temperature controlled by a Carry Peltier thermostat at 25 °C. The CF leakage measurements were performed under constant stirring. CF emission was continuously recorded in time (one measurement per second), λ_exc_ = 490 nm and λ_em_ = 512 nm. An amount of 0.1 mol % of CTX in relation to the lipid concentration was added to the LUV dispersions at the 100^th^ s. Around 10 μL of 10% (w:v) of Triton X-100 was added at the 2000^th^ s, to promote full CF leakage.

The percentage of CF leakage, (%) leakage, was determined according to:

2 $$ \left( \% \right){\text{ leakage}}\left( t \right) \, = \, \left[ {\left( {I\left( t \right) \, - I_{0} } \right) \, / \, \left( {I_{{{\text{total}}}} - I_{0} } \right)} \right] \, \times \, 100 $$

where *I*(*t*) is the fluorescence intensity at time *t*, *I*_0_ is the initial fluorescence, before CTX addition, and *I*_total_ is the maximum fluorescence obtained after the addition of Triton X-100^36,37,60^.

Kinetic analysis was performed with vesicles of DPPC, DPPG and DPPS, without and with addition of 0.1 mol% of CTX, in the lipid gel phase only (at 25 °C). The experimental procedure in the fluid phase for these lipids (at 50 °C) was considered unreliable, since a considerable leakage of CF was detected for pure lipid vesicles^36,37^. Hence, in order to mimic the fluid phase of the dipalmitoyl membranes, similarly prepared vesicles of POPC, POPG and POPS where used, without and with addition of CTX, at 25 °C.

### THP-1-macrophage differentiated preparation

They were utilized THP-1 cells, a human monocytic cell line derived from an acute monocytic leukemia patient^61^ from the Cells Bank of the Rio de Janeiro State, in Rio de Janeiro, Brazil. THP-1 cells (1 × 10^5^/mL) were cultured in RPMI medium containing fetal bovine serum (FBS) at 10% and L-glutamine 1% in an oven at 37 °C and 5% CO_2_ for 120 h. The subculture of the cells was performed every 2 days. Afterwards, the cells were collected, centrifuged and re-suspended in the fresh culture medium. To obtain THP-1-macrophage differentiated, these cells (1 × 10^6^/well) in 6-well culture plates were incubated with phorbol 12-myristate 13-acetate (PMA) (100 nM) from Sigma Chemical Co. (St. Louis, MO, USA), for 2 days. After this period, these cells were cultured in RPMI medium containing fetal bovine serum (FBS) at 10% and L-glutamine 1% in the oven at 37 °C and 5% CO_2_ for 72 h. The cells were then used in the experimental assays.

### Toxin labeling assay

Approximately 50 µg of purified toxin was resuspended in 150 µL of 10 nM HEPES, pH 7.0. The pH of the HEPES buffer was also adjusted to 8.3 by the addition of sodium bicarbonate for further toxin washes. 250 µL of fluorescein isothiocyanate (FITC) was added to the toxin solution. The reaction was carried out in the dark at room temperature for 3 h under continuous stirring and was stopped by the addition of 50 mM ammonium chloride. Excess dye was removed by Vivaspin 10 kDa column (GE Healthca®e; Little Chalfont, UK) centrifugation with 10 nM HEPES washes, pH 7.0. The conjugate was collected after washing, leaving about 20µL of labeled toxin at approximately 20 µg^62^.

#### Fluorescence assay of THP-1 cells incubated with labeled crotoxin

Differentiated macrophages from THP-1 (2.5 × 10^6^ cells/well) glass coverslips were adhered to 24-well plates for 2 h. Following the adhesion period of these cells, the cells were incubated with FITC-labeled CTX at different times. For fixation of these cells, phosphate buffered saline (1 × concentrated PBS) washing was performed and then 1 mL of permeabilizing solution (PFA + Sucrose + Triton X 100) was added for 5 min. This solution was withdrawn so that 1 mL of fixative solution containing PFA and sucrose could be added to each well for 15 min. After washing, 70 µL / well of Rhodamine Phalloidin (1: 500) was added to show actin filaments, and DAPI (4',6'-diamindini-2-phenylindole) (1: 500) for nucleus visualization by 30 min at room temperature. Then the coverslips were mounted with Vectashield, subsequently analyzed in confocal microscopy under the Leica TCS SP8-Confocal Microscope at 40 × magnification.

## Supplementary Information

Supplementary Information.
