# Supplementary material for: Correlating biological activity to thermo-structural analysis of the interaction of CTX with synthetic models of macrophage membranes
Source: Sci Rep. 2021 Dec 9;11:23712. doi: 10.1038/s41598-021-02552-0 (PMC8660830; doi:10.1038/s41598-021-02552-0)
Supplement: Supplementary file 1 — Supplementary Information. [file 41598_2021_2552_MOESM1_ESM.pdf]

Supplementary material

“Correlating Biological Activity to Thermo-Structural Analysis of the Interaction of CTX with Synthetic Models of Macrophage Membranes”

L. A. Pimenta, E. L. Duarte, G. S. V. Muniz, K. F. M. Pasqualoto, M. R. M. Fontes, M. T. Lamy, S. C. Sampaio

Table SM1. The average diameter (Z-average) and polydispersity obtained from dynamic light scattering.

Sample	Z-average (nm)	Polydispersity (%)
DPPG	113±1	6
DPPG + CTX	117±1	5
DPPS	122±1	11
DPPS + CTX	123±1	10

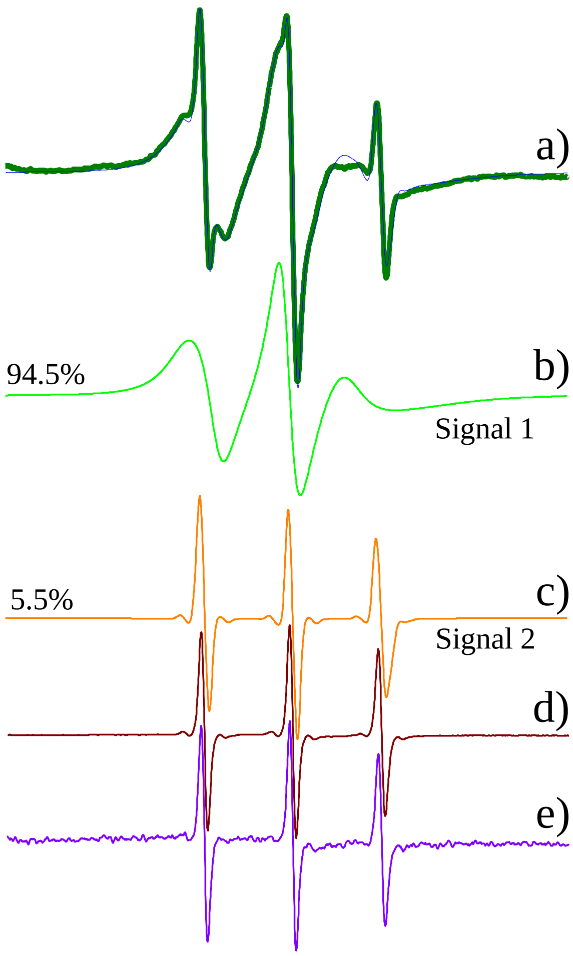

Figure SM1. ESR experimental spectrum of the 16-PCSL incorporated in gel membrane of DPPS (5mM) in the presence of 0.1 mol% CTX (a, red line), the same spectrum shown in Fig. 5l. The blue line corresponds to the best fitting of the theoretical simulation on the experimental data considering two components: the anisotropic site (b, green line) and a highly isotropic site (c, orange line. The proportion of the two components are indicated. The theoretical mobile component is very similar to the experimental spectrum obtained for 16-PCSL in solution in presence of the Crotoxin (d, wine line) and that of the pure probe 16-SASL in buffer PBS (e, violet line). The total spectra width is 100G.

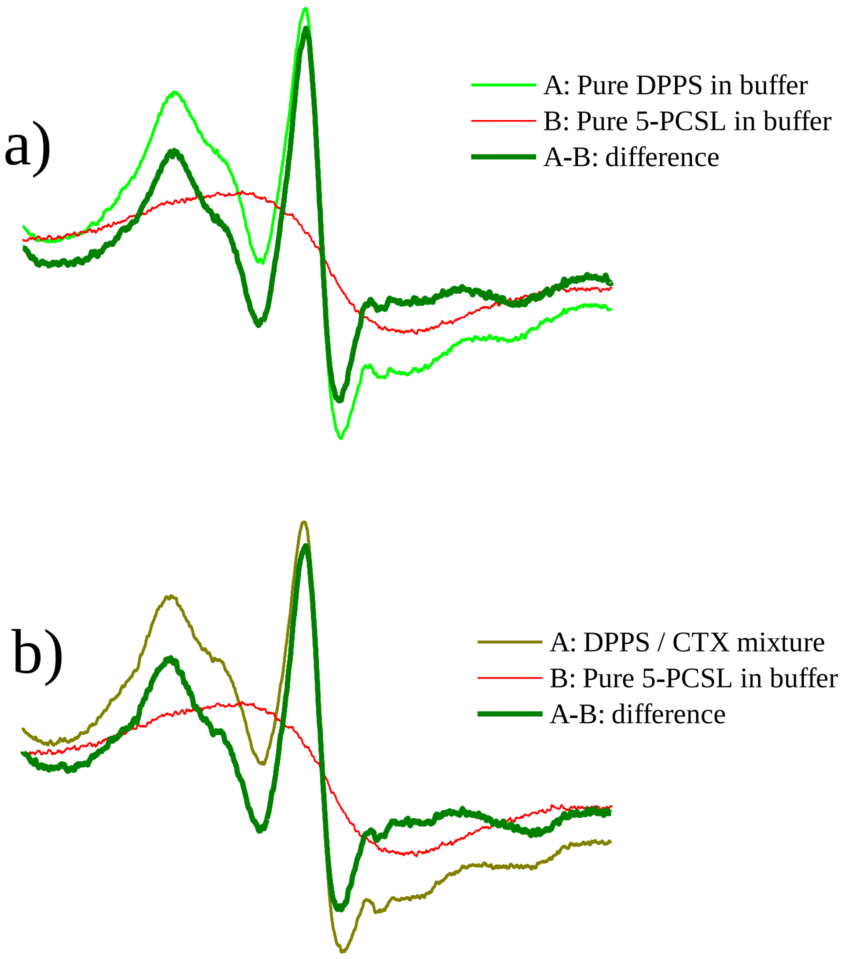

Figure SM2. ESR spectra of: (a) 5-PCSL incorporated in pure lipid bilayer of DPPS (A – green line), pure 5-PCSL in buffer PBS (B – red line) and the difference between them (A-B – olive line); and (b) 5-PCSL incorporated in the lipid bilayer of DPPS in presence of CTX (A – dark yellow line), pure 5-PCSL in buffer PBS (B – red line) and the difference between them (A-B – olive line). The lipid concentration was 5 mM in PBS buffer, 0.8 mol% of spin label and 0.1 mol% of CTX, relative to de lipid concentration was used. All spectra were acquired at the same experimental conditions, temperature of 20 ^{o}C and its total width is 100G.
